# Antiviral Activity of CD437 Against Mumps Virus

**DOI:** 10.3389/fmicb.2021.751909

**Published:** 2021-11-16

**Authors:** Fumihiro Kato, Yuichiro Nakatsu, Keiko Murano, Aika Wakata, Toru Kubota, Takayuki Hishiki, Toshiyuki Yamaji, Minoru Kidokoro, Hiroshi Katoh, Makoto Takeda

**Affiliations:** ^1^Department of Virology III, National Institute of Infectious Diseases, Tokyo, Japan; ^2^Department of Microbiology, Kanagawa Prefectural Institute of Public Health, Chigasaki, Japan; ^3^Department of Biochemistry and Cell Biology, National Institute of Infectious Diseases, Tokyo, Japan; ^4^Department of Quality Assurance, Radiological Safety, and Information Management, National Institute of Infectious Diseases, Tokyo, Japan

**Keywords:** CD437, mumps virus, knockout cell, screening, antiviral compound

## Abstract

Many efforts have been dedicated to the discovery of antiviral drug candidates against the mumps virus (MuV); however, no specific drug has yet been approved. The development of efficient screening methods is a key factor for the discovery of antiviral candidates. In this study, we evaluated a screening method using an *Aequorea coerulescens* green fluorescent protein-expressing MuV infectious molecular clone. The application of this system to screen for active compounds against MuV replication revealed that CD437, a retinoid acid receptor agonist, has anti-MuV activity. The point of antiviral action was a late step(s) in the MuV life cycle. The replication of other paramyxoviruses was also inhibited by CD437. The induction of retinoic acid-inducible gene (RIG)-I expression is a reported mechanism for the antiviral activity of retinoids, but our results indicated that CD437 did not stimulate RIG-I expression. Indeed, we observed antiviral activity despite the absence of RIG-I, suggesting that CD437 antiviral activity does not require RIG-I induction.

## Introduction

Mumps virus (MuV), of the genus *Orthorubulavirus* in the family *Paramyxoviridae*, has a worldwide circulation. Infection by MuV usually causes a mild or moderate disease with fever and swelling of the parotid gland, but is occasionally associated with serious complications such as aseptic meningitis and hearing loss (Hviid et al., 2008). Mumps vaccines are effective against MuV infection and are widely used, especially in developed countries. However, recurrent outbreaks have occurred in several countries despite their common use of mumps vaccines (Watson-Creed et al., 2006; Kutty et al., 2014; Rubin et al., 2016; Su et al., 2020). Several candidates have been reported in the development of antiviral compounds against MuV infection (McCammon and Riesser, 1979; Kosugi et al., 1994; Katoh et al., 2015; Forgione et al., 2020; Lawson et al., 2020), but no drug against MuV infection has yet been approved. Therefore, treatment for MuV infection mainly focuses on alleviating the symptoms (McLean et al., 1964; Trojian et al., 2009).

Mumps virus has a single-stranded negative-sense RNA genome of approximately 15 kb. The genome encodes seven viral proteins (N, V, P, M, F, SH, HN, and L) and possesses control regions at both genomic termini (Whelan et al., 2004; Rubin et al., 2015). In its lifecycle, the paramyxovirus attaches to the host cells, and the ribonucleoprotein (RNP) complex in the virion is released into the host cell cytoplasm. Then, the RNA genome in the RNP complex is transcribed into viral mRNAs, and translated viral proteins are used for viral genome amplification and the assembly of progeny viruses. Recently, the monitoring activities of reporter proteins encoded in recombinant virus genomes have led to the development of several high-throughput screening methods of antiviral drugs and the discovery of novel antiviral candidates (Peng et al., 2007; Tran et al., 2013; Kato and Hishiki, 2016). These reporter-based methods have several benefits, such as high sensitivity and highly efficient screening; furthermore, they have the advantage of targeting all steps, rather than a single specific step, of the viral lifecycle.

We previously developed an *Aequorea coerulescens* green fluorescent protein (AcGFP)-expressing MuV based on the Odate strain (referred to as AcGFP-MuV in this manuscript) (Katoh et al., 2017). In the present study, we evaluated whether this recombinant virus can be used in a high-throughput screening system for identifying anti-MuV drug candidates. We also evaluated the point of action of the identified anti-MuV drug candidate.

## Materials and Methods

### Construction of Knockout A549 Cell Lines

A549 cells constitutively expressing human signaling lymphocytic activation molecule (A549/hSLAM) (Takeda et al., 2005) were studied because this cell line is useful for examining not only MuV but also measles virus (MeV) as another important human paramyxovirus. To produce knockout A549/hSLAM cell lines by genome editing technology, the pSELECT-CRISPR-Cas9 plasmid was used (Ogawa et al., 2018; Yamaji et al., 2019a,b). This plasmid was cleaved by *Bsm*BI and a 20-nucleotide guide sequence against interferon-alpha/beta receptor subunit 1 (IFNAR1), mitochondrial antiviral signaling protein (MAVS), retinoic acid inducible gene I (RIG-I), or melanoma differentiation-associated gene 5 (MDA5) was inserted into this site. Guide RNA sequences were: IFNAR1: 5″-AACAGGAGCGATGAGTCTGT-3′, MAVS: 5′-TCAGCCCTCTGACCTCCAGC-3′, RIG-I: 5′-AAACAACAAGGGCCCAATGG-3′, and MDA5: 5′-CG TCTTGGATAAGTGCATGG-3′.

These plasmids were transfected into A549/hSLAM cells using TransIT-LT1 reagent (Mirus Bio, Madison, WI, United States) according to the manufacturer’s instructions. After 24 h, puromycin was added to the culture medium at a final concentration of 5 μg/ml to remove untransfected cells. Three days after puromycin addition, the culture medium was changed to puromycin-free medium, and the cells were cloned by limiting dilution. Genome sequences of the knockout cell lines were checked using Big Dye Terminator v3.1, and analyzed on an ABI3500xl Genetic Analyzer (Applied Biosystems, United States). The target knockouts were confirmed by western blotting (Supplementary Figure 1), and its method is described below.

### Cells and Viruses

Vero, VeroE6, Huh7, Hep2, A549/hSLAM, and knockout A549/hSLAM cell lines (IFNAR1-, RIG- I-, MDA5-, and MAVS-KO A549/hSLAM cells) were cultured in Dulbecco’s modified essential medium (D-MEM) (FUJIFILM Wako Pure Chemical Corporation, Osaka, Japan) with 10% fetal bovine serum (FBS), and 100 U/100 μg/ml penicillin–streptomycin (Nacalai Tesque Co., Kyoto, Japan). VeroE6/TMPRSS2 (Nao et al., 2019) and Vero/hSLAM cells (Ono et al., 2001) were cultured in D-MEM with 10% FBS, penicillin–streptomycin, and 500 μg/mL G-418 (Nacalai Tesque Co.). T7 polymerase expression BHK-21 cells (BHK/T7-9) (Ito et al., 2003) were cultured in Glasgow’s MEM (Thermo Fisher Scientific, Waltham, MA, United States) with 10% FBS and, penicillin-streptomycin.

Molecular clones of the original wild-type MuV Odate strain and AcGFP-MuV have been established in previous studies (Katoh et al., 2016, 2017). Green fluorescent protein (GFP)-expressing respiratory syncytial virus (RSV), whose details will be published elsewhere, was kindly provided by Dr. M. A. Rameix-Welti (Paris-Saclay University, INSERM, Paris, France) and Dr. J. F. Eleouet (Paris-Saclay University, INRAe, Paris, France) (Rameix-Welti et al., 2014). Green fluorescent protein-expressing human parainfluenza virus type 1 (GFP-HPIV1) was kindly provided from the National Institute of Allergy and Infectious Diseases (Rockville, MD, United States). GFP-HPIV3 was purchased from ViraTree (Research Triangle Park, NC, United States). EGFP-MeV was reported previously (Hashimoto et al., 2002). The clinical isolated MeV IC-B strain (Kobune et al., 1990), HPIV1 2272-Yamagata-2009 strain (Abe et al., 2013), and RSV A/NIID/2367/14 strain (Shirato et al., 2021) were used in the present study. Recombinant MuV was amplified using Vero cells. Recombinant RSV, recombinant and the clinical isolated HPIV1, and recombinant and the clinical isolated HPIV3 were amplified using VeroE6/TMPRSS2 cells. The clinical isolated RSV was amplified using the Hep2 cells. Recombinant and the clinical isolated MeV were amplified using Vero/hSLAM cells. Infectious viral titers were measured by plaque assays.

### Plaque Assay

Cells were seeded in 6-well plates at 1 × 10^6^ cells per well and infected with serially diluted virus samples. At 1 h post-infection (p.i.) Eagle’s minimum essential medium (E-MEM) supplemented with 2% FBS and 0.5% agarose ME was added, and the cells were incubated for 4–6 days. They were then stained with 0.033% neutral red (NR) in E-MEM and fixed with 1% glutaraldehyde in phosphate-buffered saline (PBS). If plaques were poorly visible after NR staining in reporter gene expressing recombinant viruses, the numbers were counted using a fluorescent microscope, as the plaques expressed AcGFP, EGFP, or GFP. Vero and Vero/hSLAM cells were used for MuV and MeV, respectively, VeroE6/TMPRSS2 cells were used for HPIV1, HPIV3, and recombinant RSV, and Hep2 cells were used for the clinical isolated RSV to perform the plaque assays.

### Evaluation of Screening Methods and Screening of a Chemical Library

VeroE6, Huh7, A549/hSLAM, and A549/hSLAM IFNAR1 KO cells were seeded in 96-well plates at 3 × 10^4^ cells per well. After 24 h, the cells were infected with AcGFP-MuV at a multiplicity of infection (MOI) of 0.01, then cultured with 1:20 dilution of neutralizing antibody, 10 μM (final concentration) of mycophenolic acid (MPA; Sigma-Aldrich, St Louis, MO, United States) or nocodazole (R&D Systems Minneapolis, MN, United States), which inhibits a late step of MuV infection (Katoh et al., 2015) at 37°C with 5% CO_2_. At 3 days p.i., the infection level was evaluated by observation with a fluorescence microscope (RX-800, Keyence Co., Osaka, Japan). The AcGFP brightness was analyzed using BZ-X800 analyzer software (Keyence Co.). Each test was performed 60 times. The Z′ factor was calculated using the formula described previously (Zhang et al., 1999; Kato et al., 2019).

To identify the novel anti-MuV compounds, Tocriscreen small molecule compound libraries (#2890, R&D Systems), consisting of 1,120 biologically active compounds were screened for their ability to inhibit MuV infection. A549/hSLAM IFNAR1 KO cells were seeded in 96-well plates at 3 × 10^4^ cells per well. After 24 h, EGFP-MuV at a MOI of 0.01 was inoculated and each compound was added at a final concentration of 10 μM. Cells were cultured at 37°C with 5% CO_2_ for 3 days, then examined under a fluorescence microscope. The GFP brightness was analyzed using BZ-X800 analyzer software. Additional compounds were purchased from Sigma-Aldrich.

To exclude cytotoxicity caused by the compound, a cytotoxicity assay was performed. Cells were seeded in 96-well plates at 3 × 10^4^ cells per well. After 3 days of cultivation with 10 μM of each compound, Cell Count Reagent SF (Nacalai Tesque) was used according to the manufacturer’s protocol. The absorbance at 450 nm for each well was measured using a GloMax Discover Microplate Reader (Promega, Madison, WI, United States). Each screening was performed twice.

### Calculation of the Half Maximal Effective Concentration (EC_50_) and Cytotoxicity Concentration (CC_50_)

Cells were seeded in 24-well plates at 3 × 10^5^ cells per well. Following 18 h incubation, they were infected with the viruses at a MOI of 0.01 for 1 h, and then a fresh medium containing 0–10 μM of CD437, BMS961, and CD1530 was added. After 3 days co-culture, the supernatant was collected and the viral titer was measured by plaque assay as described above. The half maximal effective concentration (EC_50_) was calculated by the Reed and Muench method (Reed and Muench, 1938).

Cells were seeded in 96-well plates at 3 × 10^4^ cells per well. After 3 days of cultivation with different concentrations of each compound (0–100 μM), Cell Count Reagent SF (Nacalai Tesque) was used according to the manufacturer’s protocol. The absorbance at 450 nm for each well was measured using a GloMax Discover Microplate Reader (Promega). The half-maximal cytotoxicity concentration (CC_50_) was then calculated.

### Time-of-Addition Assay

A549 IFNAR1 KO cells were seeded in 12-well plates at 3 × 10^5^ cells per well. After 18 h incubation, the cells were infected with AcGFP-MuV at a MOI of 2 for 1 h. Next the viral inoculum was removed, and cells were washed twice with PBS. Then, 10 μM of CD437 was added to infected cells at 2 h intervals until 22 h. After 24 h of infection, the supernatant was collected to include MuV RNA in released progeny virions; the viral titer was measured by plaque assay and MuV RNA expression was determined by quantitative reverse transcription (qRT)-polymerase chain reaction (PCR).

### Minigenome Assay

Minigenome assay was basically constructed in previous study (Katoh et al., 2017). In this study, expression vectors for N, P, and L were rearranged to pSITE vector. Expression vectors for N, P, and L MuV proteins and a luciferase coding minigenome vector were transfected into BHK/T7-9 using TransIT LT1 (Takara Bio Co., Shiga, Japan) according to the manufacturer’s instructions. Then, 0–10 μM of CD437 and 10 μM of MPA were added and incubated for 2 days. Cells were lysed, and the luciferase activity was measured using a luciferase assay system (Promega) in a microplate reader.

### Quantitative Reverse Transcription-Polymerase Chain Reaction

Total RNA was extracted from the cells and culture supernatants using a RNeasy Mini Kit (Qiagen, Germantown, MD, United States) according to the manufacturer’s protocol. MuV genome quantification has been described previously (Katoh et al., 2017). For RIG-I mRNA quantification, A549/hSLAM cells were treated with 0–10 μM of CD437 and 1,000 Units/ml of IFN αA/D (Sigma-Aldrich) for 24 h. Total RNA was extracted and one-step quantitative RT-PCR was performed using the RNA-direct SYBR Green Realtime PCR Master Mix (Toyobo, Osaka, Japan) in a LightCycler 480 system using the following primers: 5′-CTTTTTCTCAAGTTCCTGTTGGA-3′ and 5′-TCCCAACTTTCAATGGCTTC-3′ (Hergovits et al., 2017). RNA expression was normalized to that of hypoxanthine phosphoribosyltransferase 1 using the following primers: 5′-CATTATGCTGAGGATTTGGAAAGG-3′ and 5′-CTTGAGCACACAGAGGGTACA-3′.

### Western Blotting

IFNAR1-KO A549/hSLAM cell lines were seeded in 6 well plate 1 × 10^6^ cells/well and infected with MuV at a MOI of 2. After 24 h of cultivation with different concentrations of each compound (0–10 μM), the cells were lysed using Passive Lysis Buffer (Promega). The anti-MuV N rabbit polyclonal antibody was prepared as described previously (Katoh et al., 2019), and the anti-actin (AC-15) mouse monoclonal antibody was purchased from Sigma-Aldrich. Cells were collected using Passive Lysis Buffer (Promega), and protein samples were separated by sodium dodecyl sulfate–polyacrylamide gel electrophoresis and transferred to polyvinylidene fluoride blotting membranes. Bands were detected using the SuperSignal West Femto Maximum Sensitivity Substrate (Thermo Fisher Scientific). MuV N protein density was normalized to β-actin levels in the same sample. Each experiment was performed in triplicate and an average was obtained.

Knocking out IFNAR1, RIG-I, MDA5, and MAVS of A549/hSLAM were checked by western blotting (Supplementary Figure 1). RIG-I and MDA5 knockout cells were stimulated using 1,000 units/ml of IFN αA/D (Sigma-Aldrich) for 24 h. Anti-IFNAR1 (EPR6244) (Abcam, Cambridge, United Kingdom), anti-MAVS (D5A9E), RIG-I (D14G6), and MDA5 (D74E4) (Cell Signaling Technology, MA, United States) were used for detection. Can Get Signal reagent (Toyobo, Osaka, Japan) was used to enhance the signal.

### Flow Cytometry

IFNAR1-KO A549/hSLAM cell lines were seeded in 6 well plate 1 × 10^6^ cells per well and infected with MuV at a MOI of 2. After 18 h of cultivation with different concentrations of CD437 (mock, 1, 3, and 10 μM), the cells were harvested using 0.25% trypsin (Nacalai Tesque Co.). The cells were fixed and re-suspended using Flow Cytometry Fixation Buffer and Flow Cytometry Staining Buffer (R and D systems, MN, United States), and GFP expression was measured using BD FACSLyric (Becton, Dickinson and Company, NJ, United States).

### Statistical Analysis

Data are expressed as means and standard deviations (SD). All statistical analyses were performed using Student’s *t*-test; *P* < 0.05 was considered statistically significant.

## Results

### Establishment of a Screening Method for Anti-mumps Virus Candidate Drugs

**FIGURE 1:**
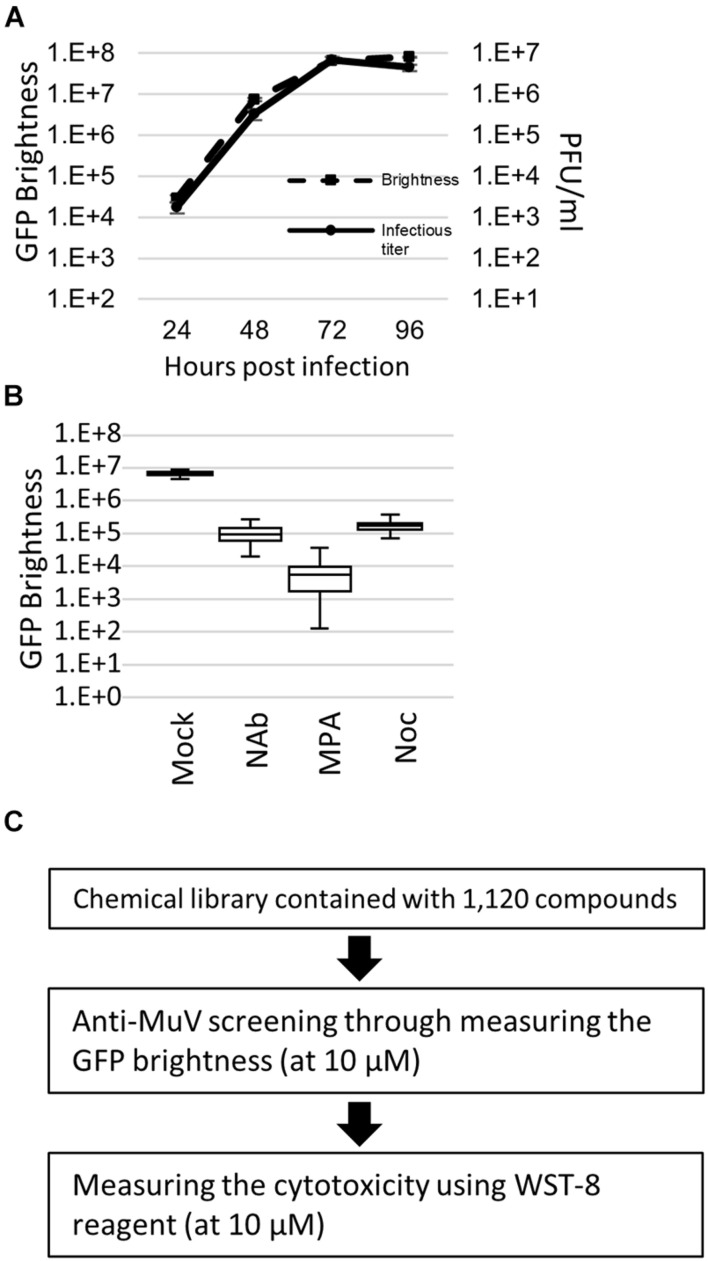
Green fluorescent protein (GFP)-expressing recombinant mumps virus (MuV)-based high-throughput screening. **(A)** Growth kinetics of the brightness and infectious virus titer. AcGFP-MuV was infected with an multiplicity of infection (MOI) of 0.01 to IFNAR1-KO A549/hSLAM cell. Data were analyzed by plaque assay in triplicate. **(B)** Validation of Z factor by measuring the GFP brightness of AcGFP Odate infected IFNAR1-KO A549/hSLAM cell at 72 h post-infection. MOI equals 0.01. Inhibitor, 1:20 diluent of neutralizing antibody, 10 μM of MPA and 10 μM of Nocodazole, were co-cultured for 3 days. Each assay was repeated 60 times. Vertical axis indicates GFP brightness (Log_10_). **(C)** Workflow of the screening in the present study. 1,120 compounds were examined, with 10 μM of the final concentration in each compound was used at the primary and secondary screening. Each screening was performed twice.

To establish an efficient screening system using AcGFP-MuV, Huh7, Vero, A549/hSLAM, and A549/hSLAM IFNAR1 KO cells were evaluated by Z′ factor, which is an important index for screening (Zhang et al., 1999). The growth kinetics based on the virus infectious titer were almost parallel to the GFP brightness measured by fluorescence (Figure 1A). Thus, virus replication was assessed by quantifying the AcGFP expression levels. MPA, which inhibits RNA synthesis in MuV and several other viruses (Kosugi et al., 1994; Diamond et al., 2002; Shen et al., 2019), was used as a control drug to evaluate the Z′ factor. The Z′ factor values for Huh7, Vero, A549/hSLAM, and A549/hSLAM IFNAR1 KO cells were 0.27, 0.67, 0.40, and 0.58, respectively. Correspondingly, Vero cells showed the highest index (Z′ factor = 0.67). However, these cells derived from the African green monkey so are not the ideal choice for drug screening, whereas A549/hSLAM cells are derived from human lung epithelial cells. Therefore, they were also evaluated, despite having a lower index. In addition, the culturing period with viruses and compounds was assessed. The 72 h p.i. and culturing with compounds indicated the clearest positive/negative ratio compared to the 24, 48, and 96 h.

An assay with a Z′ factor higher than 0.5 is generally accepted as reliable (Zhang et al., 1999), andA549/hSLAM IFNAR1 KO cells showed a higher index than parental A549/hSLAM cells (0.58 > 0.40). Therefore, they were adopted as the drug screening system in the present study. To further validate the screening method using AcGFP-MuV and A549/hSLAM IFNAR1 KO cells, the Z′ factor was also evaluated using a neutralizing antibody and nocodazole (Figure 1B and Supplementary Figure 2). The Z′ factor values were 0.54 and 0.52, respectively. The coefficient of variation index was low: 0.2% in the mock-treated cells, 0.6% in the neutralizing antibody-treated cells, 0.4% in the nocodazole-treated cells, and 1.3% in the MPA-treated cells. In A549/hSLAM IFNAR1 KO cells, the replication kinetics assessed by the infectious virus titer of the original wild-type Odate MuV strain were parallel to the AcGFP expression level of AcGFP-MuV (Figure 1C). These results indicate that the AcGFP-MuV and A549/hSLAM IFNAR1 KO cell systems are reliable and useful for the high-throughput screening of anti-MuV drugs.

### Application of the Screening System and Identification of the CD437 Candidate

**FIGURE 2:**
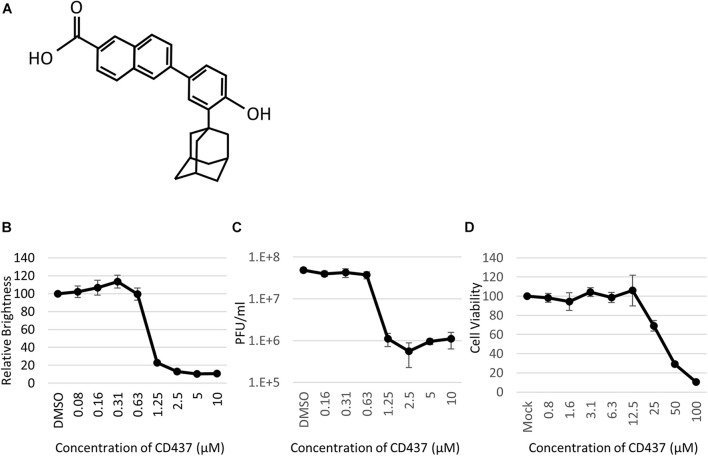
CD437 inhibited MuV replication. **(A)** Structure of CD437. **(B)** CD437 inhibited MuV replication in a dose-dependent manner. A total of 0–10 μM of CD437 was co-cultured with AcGFP-MuV at an MOI of 0.01 in IFNAR1-KO A549/hSLAM cells. At 72 h post-infection, GFP brightness was measured using a fluorescence microscope and image analyzer. EC_50_ was calculated using the Reed and Muench method. **(C)** CD437 inhibited MuV replication in a dose-dependent manner. A total of 0–10 μM of CD437 was co-cultured with AcGFP-MuV at an MOI of 0.01 in IFNAR1-KO A549/hSLAM cells. At 72 h post-infection, the supernatant was collected and the infectious titer was measured by plaque assay. The EC_50_ was calculated using the Reed and Muench method. **(D)** Cytotoxicity of CD437; 0–100 μM of CD437 was co-cultured in IFNAR1-KO A549/hSLAM cells and cell viability was measured at 72 h after culture was started using WST-8 reagent. The CC_50_ was calculated using the Reed and Muench method.

**FIGURE 3:**
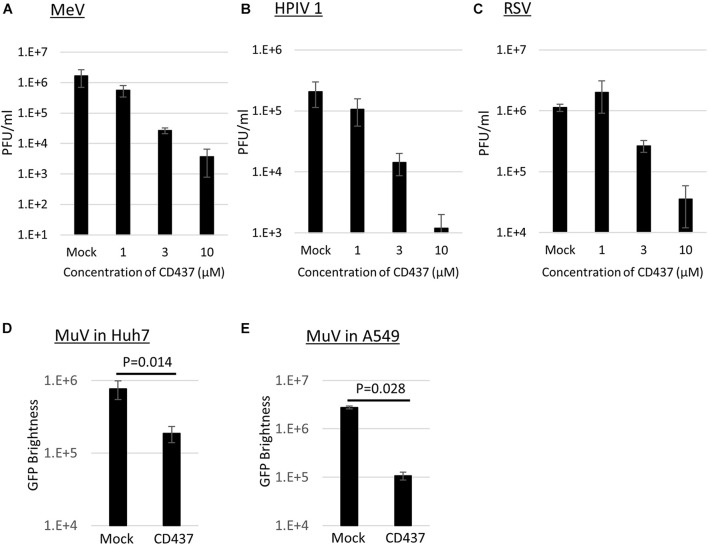
CD437 inhibition of other paramyxoviruses and MuV inhibition in Huh7 and A549 parental cells. **(A)** MeV, **(B)** HPIV1, and **(C)** RSV were inhibited by CD437. A total of 0–10 μM of CD437 was co-cultured with each virus at an MOI of 0.01. Vero/hSLAM cells were used in MeV, VeroE6/TMPRSS2 cells were used in HPIV1, and Hep2 cells were used in RSV. At 72 h post-infection, the supernatants were collected and the infectious titer was measured using a plaque assay. The EC_50_ was calculated using the Reed and Muench method. MuV was inhibited by CD437 in panel **(D)** Huh7 and **(E)** parental A547/hSLAM cells. A total of 10 μM of CD437 was co-cultured with AcGFP-MuV at an MOI of 0.01. At 72 h post-infection, GFP brightness was measured using a fluorescence microscope and image analyzer.

Anti-Mumps virus drug candidates were screened from a chemical library consisting of 1,120 compounds (Figure 1C). In the primary screening, a final concentration of 10 μM of each compound was assessed by reducing the GFP brightness signal. Next, compounds causing cell toxicity were excluded by the cytotoxicity assay as a secondary screening. The screening identified eight compounds with antiviral activity without cytotoxicity; among them, the retinoic acid receptor (RAR) gamma agonist CD437 was revealed as an anti-MuV candidate (Figure 2A). When inhibition was assessed using AcGFP expression, the EC_50_ of CD437 against MuV infection was 0.98 ± 0.02 μM (Figure 2B). As expected, a similar EC_50_ level (0.79 ± 0.11 μM) was shown when inhibition was assessed using infectious virus titers (Figure 2C). The CC_50_ of CD437 was 34.9 μM (Figure 2D), and the selective index (CC_50_/EC_50_ = 34.9/0.79) was 46.1. The anti-MuV effect of CD437 was also confirmed using Huh7 and parental A549/hSLAM cells (Figures 3E,F), indicating that the effect was unrelated to IFNAR1 KO and not specific to A549/hSLAM cells. CD437 also exhibited antiviral activity against other viruses in the family Paramyxoviridae (MeV, HPIV1, and HPIV3) and family Pneumoviridae (RSV) (Figure 3 and Supplementary Figure 3), with an EC_50_ of 0.74 at MeV, 1.16 at HPIV1, and 2.34 at RSV.

### CD437 Exhibits Anti-mumps Virus Activity Independently of the Retinoic Acid-Inducible Gene-I-Like Receptor Signaling Pathway

**FIGURE 4:**
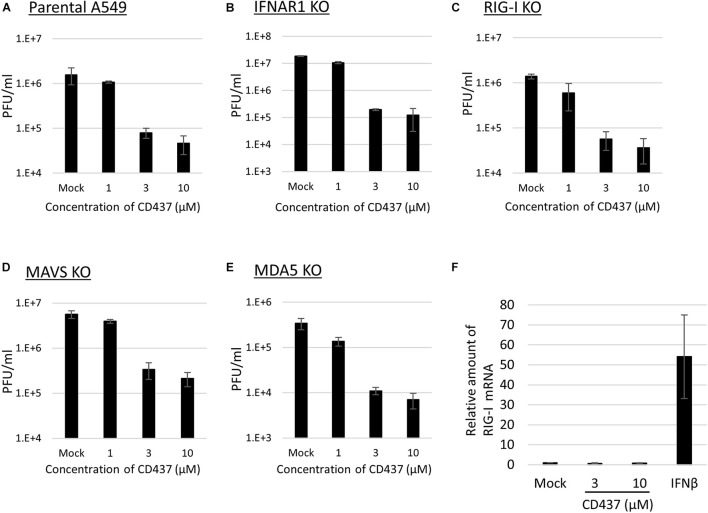
CD437 also inhibited MuV replication in RIG-I knockout cells. MuV was inhibited by CD437 in panel **(A)** parental A549/hSLAM cells, **(B)** IFNAR1-KO A549/hSLAM cells, **(C)** RIG-I-KO A549/hSLAM cells, **(D)** MAVS-KO-A549/hSLAM cells, and **(E)** MDA5-KO-A549/hSLAM cells. A total of 0–10 μM of CD437 was co-cultured with the MuV Odate strain at an MOI of 0.01. At 72 h post-infection, the supernatant was collected and the infectious titer was measured by plaque assay. The EC_50_ was calculated using the Reed and Muench method. **(F)** Quantification of retinoic acid-inducible gene (RIG-I) mRNA using IFN β as a control. A total of 0–10 μM of CD437 and 1,000 Units/ml of IFN αA/D were treated with A549/SLAM for 24 h. mRNA was measured by qRT-PCR.

A previous study revealed that CD437 functions as an agonist of RAR gamma (Bernard et al., 1992). To determine whether there is a relationship between RAR gamma agonistic activity and the antiviral activity of CD437, two RAR-related compounds (CD1530, an analog of CD437, and BMS961, an agonist of RAR gamma) were analyzed for antiviral activity against MuV. No anti-MuV activity was observed for any of the two compounds (Supplementary Figure 4). Previous studies also revealed that retinoids bind to RAR and inhibit MeV and MuV infections via RIG-I promoter activation (Soye et al., 2011, 2013). To assess the contribution of the RIG-I signaling pathway to the anti-MuV activity of CD437, similar experiments were performed using RIG- I-, MDA5-, and MAVS- KO A549/hSLAM cells. CD437 inhibition of MuV propagation was observed in RIG- I-, MDA5-, and MAVS-KO cells, as well as in IFNAR-KO and parental A549/hSLAM cells (Figures 4A–E). Consistent with these results, CD437 did not increase RIG-I mRNA expression in A549/hSLAM cells, whereas the IFNβ treatment did so significantly (Figure 4F). These results showed that the anti-MuV activity of CD437 was independent of the RIG-I signaling pathway (Figure 4F).

### CD437 Inhibits a Late Step of the Mumps Virus Multiplication Cycle

**FIGURE 5:**
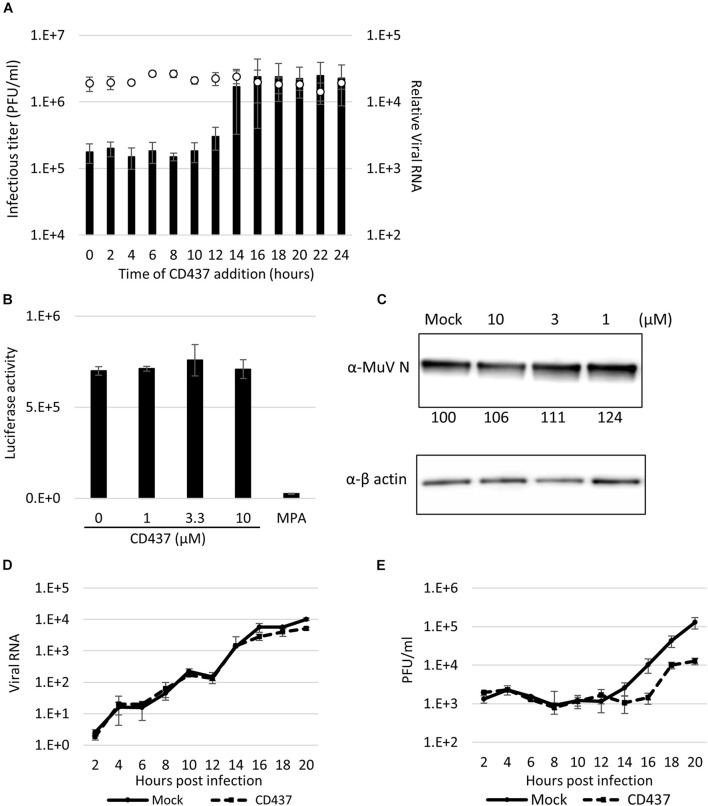
CD437 inhibited a late step in the MuV life cycle. **(A)** Time-of-addition assay. A total of 10 μM of CD437 was co-cultured with AcGFP-MuV at MOI of 2 in IFNAR1-KO A549/hSLAM cells. The left vertical axis and bar indicate the infectious titer in the supernatant 24 h post-infection. The right vertical axis and white circle indicate the intracellular RNA expression. **(B)** Minigenome assay. N, P, and L of MuV protein expression vector and luciferase coding minigenome plasmid were transfected into IFNAR1-KO A549/hSLAM cells and co-cultured with 10 μM of CD437. The luciferase activities were measured at 48 h post-transfection. **(C)** Detection of MuV N protein with or without CD437 treatment at 24 h post-infection. IFNAR1-KO A549/hSLAM cells were cultured with 10 μM or mock medium and infected with MuV at MOI of 2. Western blotting was performed and the band density was measured. **(D)** One-step growth curve of viral RNA in cells. IFNAR1-KO A549/hSLAM cells were cultured with 10 μM or mock medium and infected with MuV at MOI of 2. The cells were collected every 2 h and viral RNA was quantified by qRT-PCR. **(E)** One-step growth curve of the infectious titer in the supernatant. IFNAR1-KO A549/hSLAM cells were cultured with 10 μM or mock medium and infected with MuV at MOI of 2. The supernatants were collected every 2 h and the virus titer was quantified by plaque assay.

To identify the point of action of CD437 in the MuV cycle, a time-of-addition assay was performed. During a single MuV life cycle, during the first few hours, the virus attaches and invades the host cells, and primary transcription begins immediately, and viral proteins are translated within the next several hours. RNA synthesis begins started at 4–6 h after infection. Viral particles and genomes are assembled, and progeny virus are released in to outer cell approximately 16 h after the first infection. CD437 reduced the production of infectious viruses, even when it was added as late as 12 h p.i. (Figure 5A). Furthermore, the viral genome levels were not affected by CD437 treatment.

To clarify this further, the effect of CD437 on the MuV minigenome replication system, which specifically reproduces viral genome replication and transcription, was analyzed. CD437 did not inhibit MuV minigenome RNA synthesis at any concentration (Figure 5B). Next, at a single infection round, viral protein and GFP expression levels after CD437 treatment were analyzed by western blotting and flow cytometry, respectively, and there was no significant change after CD437 treatment (Figure 5C and Supplementary Figure 5). These results indicated that CD437 did not affect viral protein translation or GFP activity. Finally, viral RNA levels in the inner cells and progeny virus titer outer cells at a single infection round were assessed. CD437 did not affect MuV RNA levels in cells during a single round infection, especially in early steps (0–12 h p. i.); however, it significantly reduced MuV infectious titers in culture media at or after 16 h p.i., regarded as late steps (Figures 5D,E). Therefore, CD437 inhibited the late step event(s), such as assembly, budding, and releasing steps, although it did not inhibit the early viral replication steps, including the viral attachment and entry step, or the viral protein expression and genome synthesis.

## Discussion

In this study, we evaluated AcGFP-MuV for the high-throughput screening of antiviral candidates. The screening method presented in this study could identify candidates targeting any of multiple steps of the viral cycle, and evaluated viral growth quickly, easily, and sensitively. However, our system cannot identify drug candidates targeting IFN-related pathways because IFNAR1 KO cells are used.

We screened the antiviral activity of a chemical library consisting of 1,120 compounds with known pharmacological activity and a wide coverage of cellular targets (Kato et al., 2019). This confirmed that it was possible to identify candidates with novel antiviral mechanisms, and that it was relatively easy to estimate the point of action despite phenotypic screening. In fact, we identified various anti-dengue virus candidates from the same chemical library in a previous study (Kato et al., 2019). The present study identified CD437 as an anti-MuV candidate.

Recently, antiviral drug candidates with strong suppressive activity against positive- and negative-stranded RNA viruses have been identified, including remdesivir, NITD008, MPA, and favipiravir. These drugs have a broad spectrum against various viruses and can be quickly adapted for use against emerging or re-emerging diseases by drug repositioning (Barrows et al., 2016; Tani et al., 2016; Wang et al., 2020). In a previous study, CD437 demonstrated antiviral activity against SARS-CoV-2, which is a single positive-stranded RNA virus (Kato et al., 2020). CD437 has also shown antiviral activity against many paramyxo- and pneumoviruses.

Given broad antiviral spectrum was shown, the antiviral mechanism of CD437 may be related to host factors. The RAR has been reported as a key factor for antiviral activity in several candidates. Previous studies suggested that retinoid-induced RIG-I expression is essential for their antiviral activity (Soye et al., 2011, 2013). Indeed, CD437 has potent pharmacological activity as a RAR agonist. We showed that it still has antiviral activity in RIG-I KO cells, and does not increase the mRNA expression of RIG-I, suggesting that its mechanism of action does not require RIG-I and that a different novel mechanism may be responsible for its antiviral activity. Some studies have reported that CD437 induces apoptosis by increasing the cyclin-dependent kinase inhibitory factor through a RAR-independent pathway (Hsu et al., 1997; Zhao et al., 2001). In the present study, evident apoptosis was not observed after CD437 treatment, and CD437 acted as a specific point of action in the viral life cycle. Therefore, the mechanism of CD437 antiviral activity might not be related to apoptosis; however, careful experiments and discussion are needed in future studies.

CD437 did not inhibit the early steps of the MuV life cycle, such as attachment and entry, transcription, translation of viral proteins, and RNA synthesis. However, it did inhibit the later steps such as assembly, budding, and the release of progeny virus. In multi-round infections, the reduced production of the progeny virus by CD437 resulted in reduced secondary and subsequent infections and eventual antiviral activity. In the present study, we could not identify a definitive point of action during the late steps. It is also possible that there was more than one point of action in these steps. In other viruses, such as neuraminidase inhibitors in the influenza virus, late step inhibitors are one of the targets for developing antiviral drugs. Clarification of this critical point of action is important for the future development of CD437 as an antiviral drug candidate.

CD437 inhibited a late step(s) of the MuV lifecycle. Only a small number of candidates, including nocodazole, have been reported as having antiviral activity against MuV and acting in the late steps of the virus lifecycle. However, nocodazole is a toxic compound that interferes with the polymerization of microtubules and interrupts the cell cycle; therefore, there are concerns about its therapeutical use. Thus, CD437 could be an alternative antiviral candidate that suppresses a late step(s) of the MuV viral cycle.

In conclusion, this study developed an efficient high-throughput screening method for MuV antiviral drug discovery. This enabled the identification of CD437 as having anti-MuV activity, and targeting a late step(s) of the MuV replication cycle. Further studies about the detailed mechanism underlying the point of action of CD437 against MuV infection may lead to the development of novel antiviral drugs.

## Data Availability Statement

The original contributions presented in the study are included in the article/Supplementary Material, further inquiries can be directed to the corresponding author.

## Author Contributions

FK, YN, and KM performed the experiments and analyzed the data. FK, YN, and MT wrote the manuscript. TH and TY provided the reagents. KM, AW, TK, TH, TY, MK, and HK interpreted the data and edited the manuscript. All authors contributed to the article and approved the submitted version.

## Conflict of Interest

The authors declare that the research was conducted in the absence of any commercial or financial relationships that could be construed as a potential conflict of interest.

## Publisher’s Note

All claims expressed in this article are solely those of the authors and do not necessarily represent those of their affiliated organizations, or those of the publisher, the editors and the reviewers. Any product that may be evaluated in this article, or claim that may be made by its manufacturer, is not guaranteed or endorsed by the publisher.

## Supplementary Material

Supplementary Figure 1 Confirmation of knockout by western blotting. **(A)** Confirmation of IFNAR1 and MAVS knockout. A549/hSLAM, IFNAR1-KO-A549/hSLAM, and MAVS-KO-A549/hSLAM were lysed and analyzed by western blotting. **(B)** Confirmation of knockout in RIG-I and MDA5 cells. A549/hSLAM, RIG-I-KO-A549/hSLAM, and MDA5-KO-A549/hSLAM were treated with 1,000 Units/ml of IFN αA/D for 24 h. The cells were lysed and analyzed by western blotting.

Supplementary Figure 2 **(A)** Neutralizing antibody, **(B)** MPA, and **(C)** nocodazole inhibited MuV. Each reagent was diluted and co-cultured with MuV Odate strain at MOI of 0.01 in IFNAR1-KO A549/hSLAM cells. At 72 h post-infection, the supernatant was collected, and the infectious titer was measured using a plaque assay. EC_50_ was calculated using the Reed and Muench method.

Supplementary Figure 3 **(A)** MeV, **(B)** HPIV1, **(C)** HPIV3, and **(D)** RSV inhibited CD437 expression. A total of 10 μM of CD437 was co-cultured with AcGFP-MuV at MOI of 0.01. At 72 h post-infection, GFP brightness was measured using a fluorescence microscope and an image analyzer.

Supplementary Figure 4 Analogs of CD437 and other RAR agonists had no indication of antiviral activity. GFP brightness of AcGFP-MuV under treatment with **(A)** CD1530, an analog of CD437, and **(B)** BMS961, RAR gamma agonist. A total of 0–10 μM of each compound was co-cultured with AcGFP-MuV at an MOI of 0.01 in IFNAR1-KO A549/hSLAM cells. At 72 h post-infection, the brightness of GFP was measured.

Supplementary Figure 5 CD437 did not affect GFP expression. (A) Detection of GFP positive cells under treatment with CD437 by flow cytometry. (B) Percentage of GFP positive cells. AcGFP-MuV at MOI of 2 and 10 μM of CD437 or mock infected/treated cells were cultured for 18 h in IFNAR1-KO A549/hSLAM cells. GFP expression was measured using flow cytometry.
