# Supplementary figures and images for: Antiviral Activity of CD437 Against Mumps Virus
Source: Front Microbiol. 2021 Nov 16;12:751909. doi: 10.3389/fmicb.2021.751909 (PMC8636907; doi:10.3389/fmicb.2021.751909)

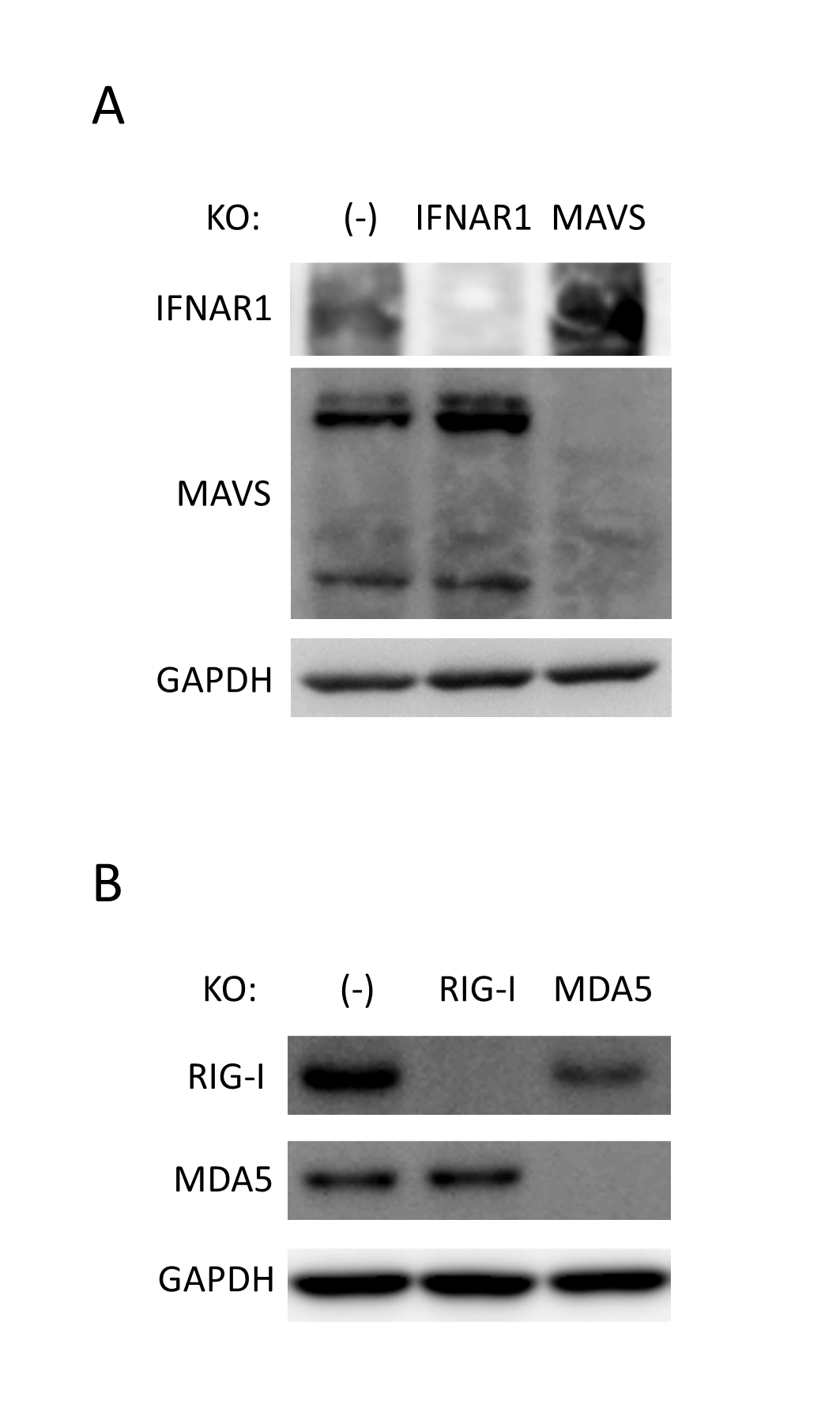

Supplement: Supplementary Figure 1 — Confirmation of knockout by western blotting. (A) Confirmation of IFNAR1 and MAVS knockout. A549/hSLAM, IFNAR1-KO-A549/hSLAM, and MAVS-KO-A549/hSLAM were lysed and analyzed by western blotting. (B) Confirmation of knockout in RIG-I and MDA5 cells. A549/hSLAM, RIG-I-KO-A549/hSLAM, and MDA5-KO-A549/hSLAM were treated with 1,000 Units/ml of IFN αA/D for 24 h. The cells were lysed and analyzed by western blotting. [file Image_1.tif]

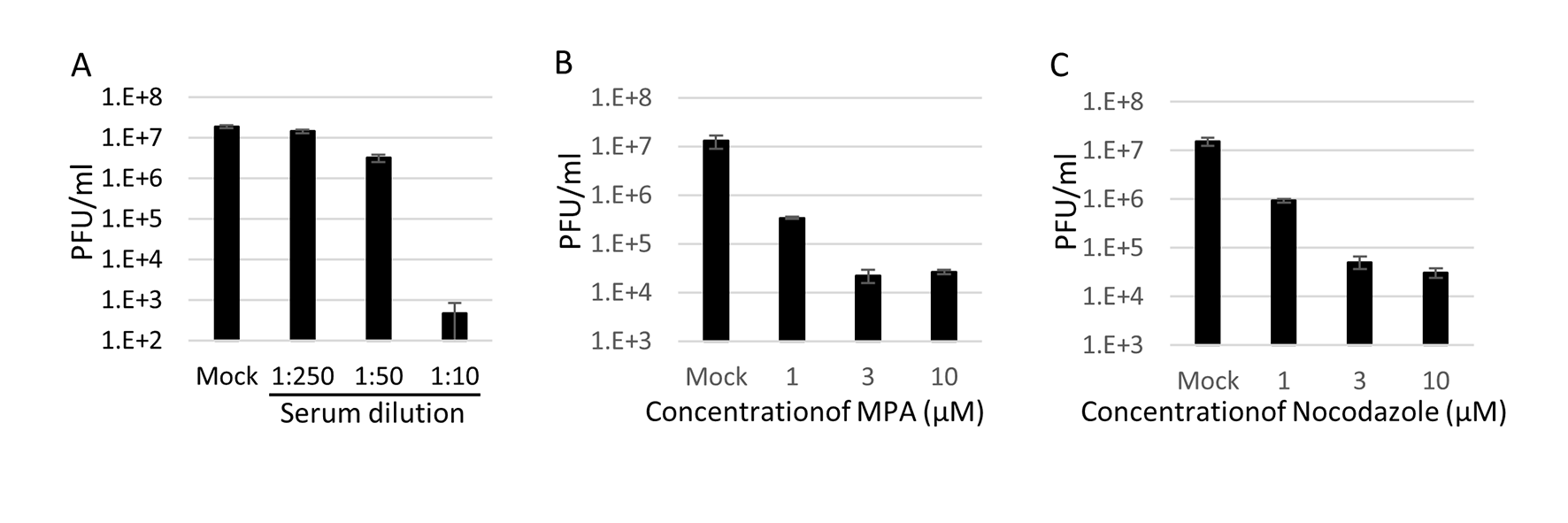

Supplement: Supplementary Figure 2 — (A) Neutralizing antibody, (B) MPA, and (C) nocodazole inhibited MuV. Each reagent was diluted and co-cultured with MuV Odate strain at MOI of 0.01 in IFNAR1-KO A549/hSLAM cells. At 72 h post-infection, the supernatant was collected, and the infectious titer was measured using a plaque assay. EC50 was calculated using the Reed and Muench method. [file Image_2.tif]

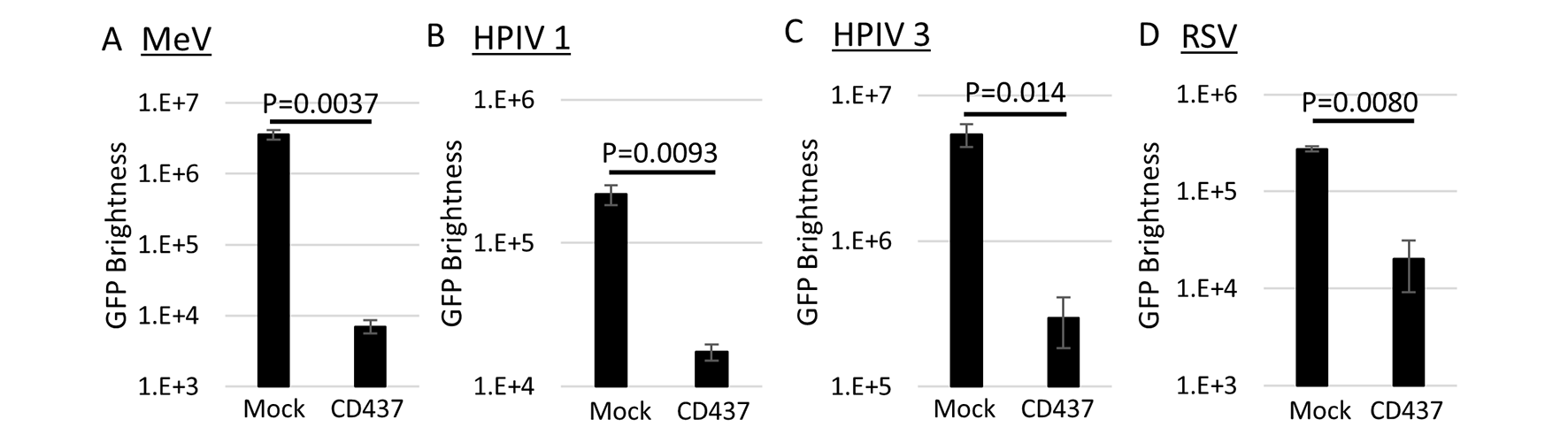

Supplement: Supplementary Figure 3 — (A) MeV, (B) HPIV1, (C) HPIV3, and (D) RSV inhibited CD437 expression. A total of 10 μM of CD437 was co-cultured with AcGFP-MuV at MOI of 0.01. At 72 h post-infection, GFP brightness was measured using a fluorescence microscope and an image analyzer. [file Image_3.tif]

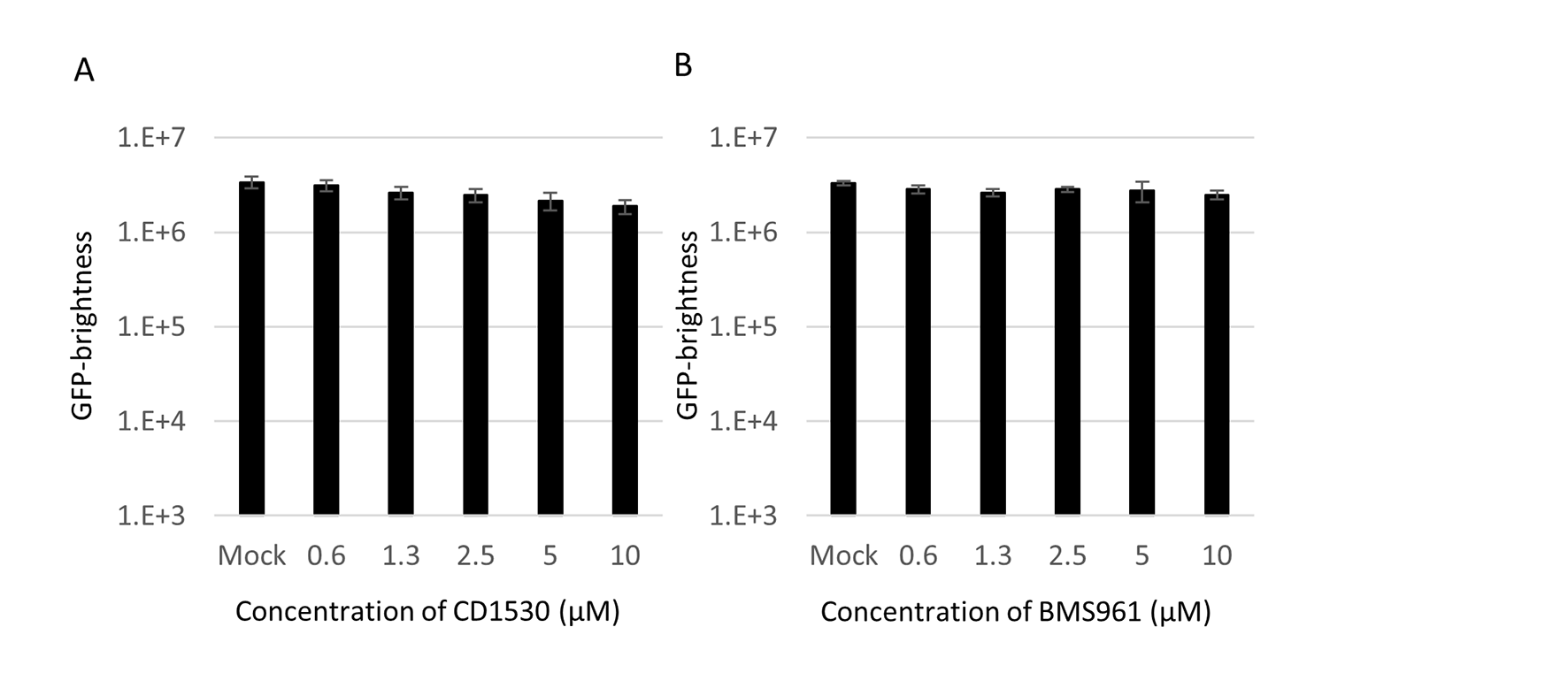

Supplement: Supplementary Figure 4 — Analogs of CD437 and other RAR agonists had no indication of antiviral activity. GFP brightness of AcGFP-MuV under treatment with (A) CD1530, an analog of CD437, and (B) BMS961, RAR gamma agonist. A total of 0–10 μM of each compound was co-cultured with AcGFP-MuV at an MOI of 0.01 in IFNAR1-KO A549/hSLAM cells. At 72 h post-infection, the brightness of GFP was measured. [file Image_4.tif]

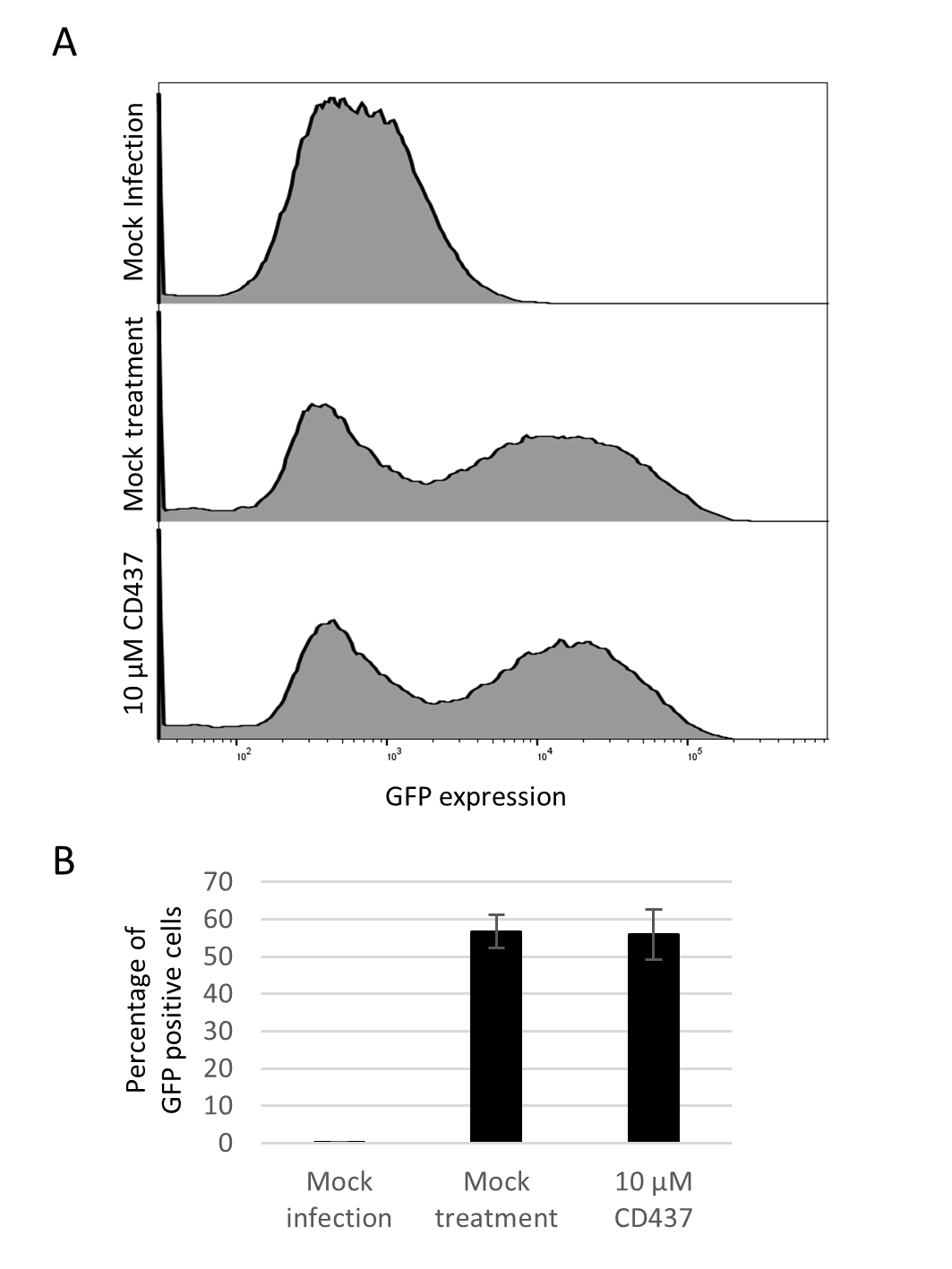

Supplement: Supplementary Figure 5 — CD437 did not affect GFP expression. (A) Detection of GFP positive cells under treatment with CD437 by flow cytometry. (B) Percentage of GFP positive cells. AcGFP-MuV at MOI of 2 and 10 μM of CD437 or mock infected/treated cells were cultured for 18 h in IFNAR1-KO A549/hSLAM cells. GFP expression was measured using flow cytometry. [file Image_5.tif]
